# Supplementary figures and images for: The Zoige pioneer plant Leymus secalinus has different endophytic bacterial community structures to adapt to environmental conditions
Source: PeerJ. 2023 May 18;11:e15363. doi: 10.7717/peerj.15363 (PMC10200098; doi:10.7717/peerj.15363)

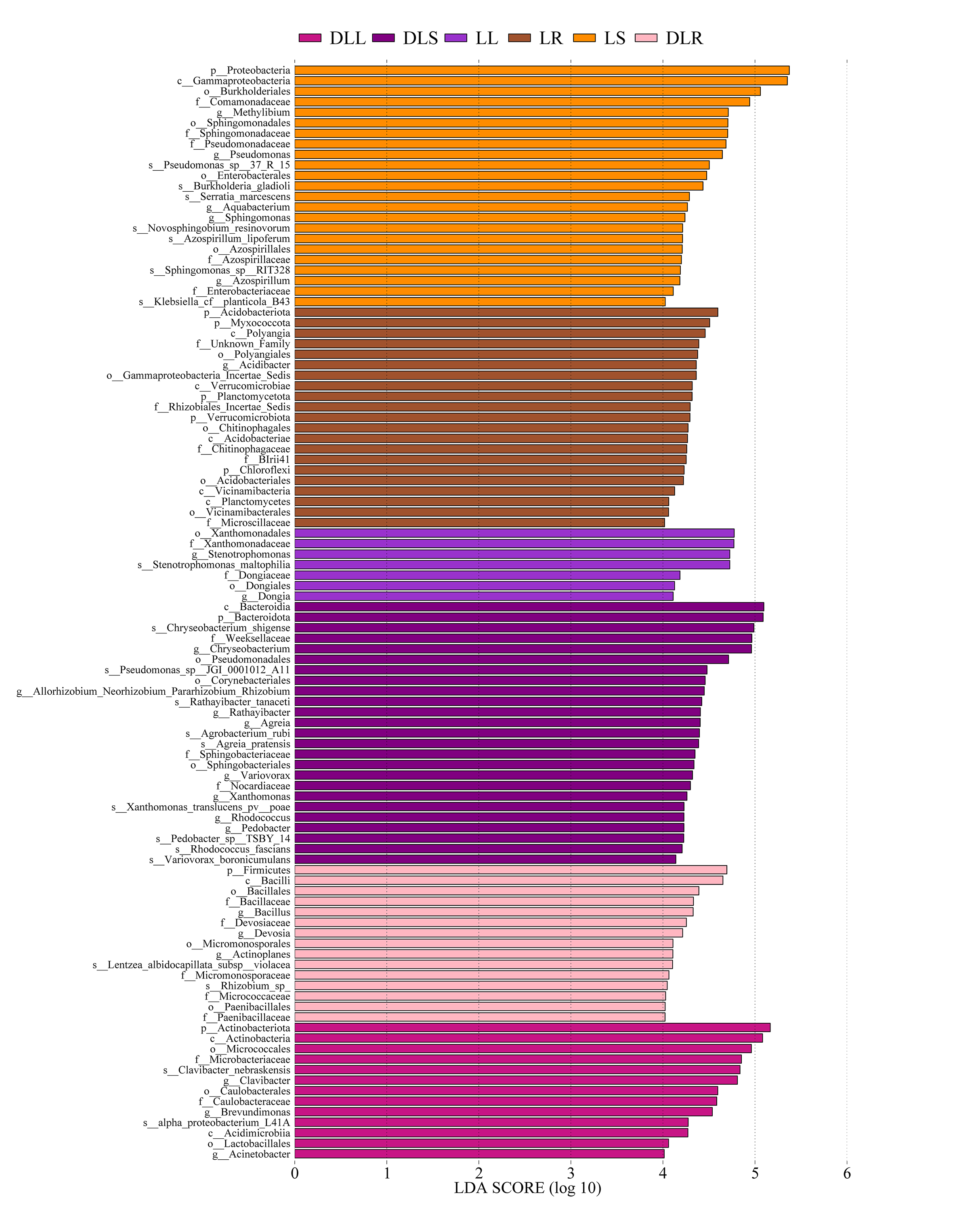

Supplement: Figure S1 [file peerj-11-15363-s001.png]

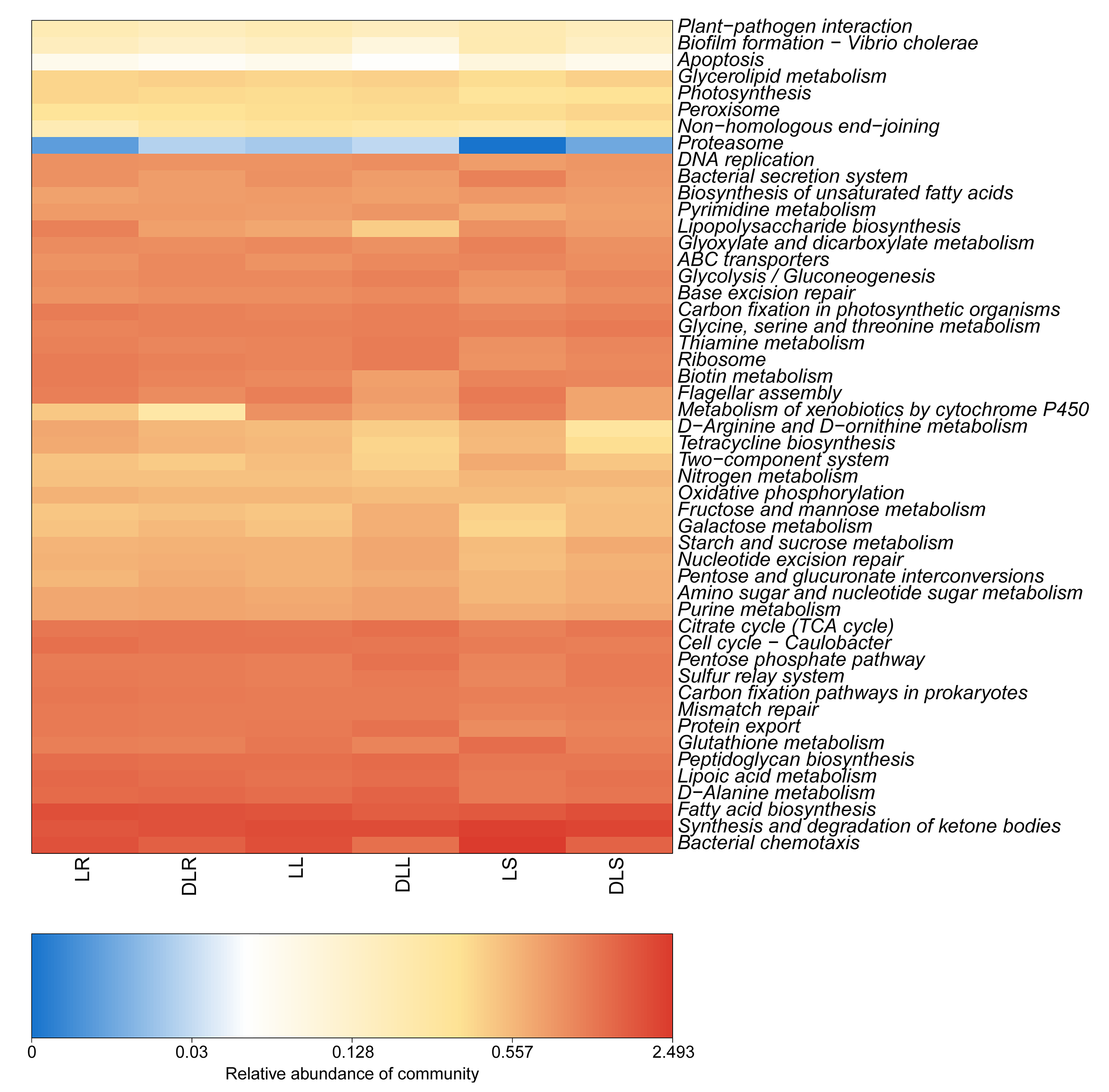

Supplement: Figure S2 [file peerj-11-15363-s002.png]
